# Determinants of academic achievement among higher education student found in low resource setting, A systematic review

**DOI:** 10.1371/journal.pone.0294585

**Published:** 2023-11-20

**Authors:** Chalachew Kassaw, Valeriia Demareva

**Affiliations:** 1 Department of psychiatry, Dilla University, Dilla, Ethiopia; 2 Faculty of Social Sciences, Lobachevsky State, University of Nizhny Novgorod, Nizhny Novgorod, Russia; 3 Department of Social Security and Humanitarian Technologies, Nizhny Novgorod State University, Nizhniy Novgorod, Russia; University of Hail, SAUDI ARABIA

## Abstract

**Background:**

Academic success is a measure of students’ ability to attain their educational objectives, often assessed through regular evaluations or examinations. To establish effective policies and programs that align with academic accomplishments, conducting comprehensive data analysis is pivotal. Hence, this systematic review aimed to synthesize the factors impeding the academic achievements of Ethiopian students in higher education.

**Methods:**

A comprehensive review was conducted on studies involving Ethiopian university students from 2013 to 2022. The review encompassed 24 papers that were gathered from different databases like PubMed, Google Scholar, African Journals Online, Scopus, and Web of Science.

**Results:**

The findings of this research revealed that inadequate classroom environments, experiencing dysmenorrhea, and engaging in excessive social media usage were all linked to a decline in academic performance. Conversely, adopting healthy sleep habits, achieving high scores in entrance exams, and avoiding recent substance abuse were all factors positively influencing academic success. In addition, there was a positive correlation between academic excellence and being a health science college student and age range of 20 to 24 years old.

**Conclusion:**

To enhance academic performance, it is crucial to address the negative factors identified, such as inadequate classroom environments, dysmenorrhea, and excessive social media usage, while promoting positive factors like healthy sleep habits, high scores in exams, and avoiding substance abuse. Additionally, being a health science college student and belonging to the age range of 20 to 24 were found to be associated with academic excellence.

## Introduction

Academic success pertains to the extent of accomplishment exhibited by a student or institution in attaining educational objectives, regardless of whether they are immediate or long-range in nature [1]. Education is a formidable catalyst for transforming a nation’s societal harmony, economic prosperity, standard of living, and overall well-being [2]. Graduation rates evaluate the performance of an institution, while GPA (Grade Point Average) measures the achievements of individual students [3]. GPA (Grade Point Average) is calculated by dividing the sum of grade points by the total units [4]. The assessment of students’ knowledge and skills attained from each subject depends on the subject-area measurement level [5].

Higher education institutions play a vital role in creating an environment that promotes learning and supports the development of global competencies in various academic disciplines. This, in turn, allows learners to effectively navigate the ever-changing global landscape. Ultimately, such efforts enhance the overall quality of education and equip students to successfully overcome challenges [6]. Learning is a lifelong and challenging process that does not guarantee the attainment of knowledge, skills, or perspectives. It requires significant effort and time [7]. In order to succeed in school, students must exhibit initiative, self-control, effective time management, focused attention, inquisitiveness, and active engagement in the classroom [8]. Good academic performance offers numerous benefits, including improved living conditions, increased productivity, and better economic prospects for society. It also provides students with a positive self-image, confidence, good mental health, social skills, and a clear vision for their future [9]. Poor academic performance in students can potentially lead to a range of psychological problems, such as substance abuse, criminal behavior, promiscuity, and conflicts in relationships [10]. There were also encounters of difficulties with timely graduation due to retakes and grade changes, strained relationships with professors and support staff, as well as conflicts with college deans and students [11]. The government’s extensive educational efforts have failed to assist many students in achieving higher academic levels [12]. The number of students enrolling in Ethiopia’s higher education institutions is not comparable to the number of graduates because a significant portion of applicants are initially rejected, then withdraw, and ultimately get readmitted [13]. Challenging situations can often result in family struggles, dependence, lack of insurance, poverty, and insufficient access to healthcare coverage [14]. The effectiveness of teaching and learning tools, along with the students’ personality, goals, and teachers’ skills, all have an impact on academic progress. Studies have shown that the environment also plays a critical role in students’ performance in school [15]. Academic achievement is influenced by several factors, including finances, study habits, time management, health, and family connections, all of which are significant [16]. Poor academic performance has been found to be linked to several factors, including sporadic school attendance, low parental education, unstable family relationships, excessive use of social media, and spending excessive amounts of time engaging in conversation [17]. Research conducted by national universities has identified specific characteristics that are consistently associated with poor academic performance [18]. For instance, a study conducted by Bahir Dar University discovered that a student’s academic status is influenced by the education level of their parents and their tendency to frequent pubs and clubs [19]. However, the results of a study at Arba Minch University showed that a student’s past academic achievement largely predicts their present performance on campus [20]. An additional examination conducted at Wolayita Sodo University discovered a correlation between present drug usage and academic achievement [21]. Currently, there are 42 public institutions in the country, all of which strive arduously to improve the quality of education [22]. Assessing the academic performance of students is crucial for ensuring quality assurance in higher education institutions. However, analyzing national averages of academic predictors is an essential tool for developing academic policies and strategies that can enhance education quality on a broader scale. This review specifically aimed to identify the main predictors of academic achievement based on studies conducted among universities located in different regions of Ethiopia. The review found that participation in a supportive academic environment, acquiring essential information, maintaining a positive outlook, and possessing subject-specific abilities are crucial factors for student success. While numerous individual studies have been conducted in various parts of the country that have identified potential factors associated with academic achievement, decision-makers who are striving to improve academic standards in Ethiopian higher education institutions will find this comprehensive review particularly beneficial. Moreover, the review also identified areas of knowledge gaps that require further exploration in order to enhance academic quality throughout the country.

## Methods

### Study design and setting

A systematic review covering studies conducted in Ethiopian higher education institutions between 2013 and 2022 was conducted between January and February of 2023. As of 2023, Ethiopia will have 83 private institutions, 42 public universities, and 677 study options. Additionally, more than 150,000 adults graduate annually in the country. The universities offer training for students pursuing undergraduate, graduate, and doctoral degrees [23]. We have checked the Prospero database (http://www.library.ucsf.edu/) to determine if there are any published or ongoing projects related to the topic, in order to avoid any duplication. The findings revealed that there are no ongoing or published articles in the area of this topic. The current systematic review followed the PRISMA (Preferred Reporting Items for Systematic Reviews and Meta-Analyses) criteria (S1 File) [24].

### Searching strategy and source of information

An extensive literature search was conducted using international databases, including PubMed, Scopus, Google Scholar, African Journals Online, and Web of Science, to retrieve relevant articles. Search terms were formulated following the Population, Intervention, Comparison, and Outcomes (PICO) framework and applied to the online databases. Medical Subject Headings (MeSH) terms and key terms were developed using various Boolean operators, such as "AND" and "OR." The following search terms were used: “Academic Achievement”, OR “Academic Performance” OR “Average Cumulative Grade Point”, OR “Performance Indicators” AND Psychological Determinants”, “Biological Determinants”, “Social Determinants”, “Higher Education”, “Competency Measures”, AND “Teaching-learning styles Predictors” AND “Ethiopia” **(S2 File).**

### Eligibility criteria

The authors performed an unbiased eligibility examination based on the provided criteria. Issues were resolved through mutual agreement and the involvement of other authors. This systematic review analyzed articles written in English, published between 2013–2022 and that investigated predictors of academic achievement among higher education students. Only studies with cross-sectional designs, defined outcome variables, and covariates were included. This research aimed to identify factors that contribute to academic success among college students. Studies that did not use basic statistical analysis (Prevalence, Mean, ANOVA, T-test, adjusted odds ratio and Crude odd ratio) to establish the connection between academic performance and its influencing factors were excluded from the review.

### Operational definitions

#### Outcome measurement (Academic achievement)

Grade Point Average (GPA): Grade point average (GPA) is a value calculated by multiplying the unit value for each course by the grade point total and then dividing the sum by the total number of units.

A checklist: An assessment tool lists the specific criteria for skills, behaviors, or attitudes that participants must demonstrate to show that they have successfully learned from training.

Writing assessment: It refers to a field of study that contains theories and practices that guide the evaluation of a writer’s performance or potential through writing tasks.

Interview assessment: An interview based test used to evaluate a student’s suitability for the particular subject they wish to pursue in a specific department.

A skills gap analysis: It is a tool used to assess the gap between a student’s current capabilities and the requirements of a particular profession, both current and future.

#### Determinant factors of the outcome measurement

Psychological factors: The internal influences shape our thoughts, feelings, and behaviors.

It includes mental pain, sleep quality, self-esteem, prosocial behavior, anxiety, depression, and suicidality.

Biological factors: These are the physical and chemical influences on our bodies and minds.

It includes gender, age, and hormonal issues (dysmenorrhea). Facility-related factors: This physical and environmental conditions support student learning. It includes availability of adequate seating and studying spaces, lighting, technology, equipment and supplies, sleeping accommodations, dining halls, sports fields, green spaces and other outdoor areas.

Life style factors: These are the choices and behaviors that people make that can affect their health and well-being. It includes excessive social media usage, premarital sex, and sexual abstinence.

#### Study selection and data extraction

The researchers used the reference management software Mendeley, Desktop and Endnote version 25 to remove duplicate articles from the search results. Three independent reviewers then screened the titles and abstracts of the remaining articles to determine eligibility for the review. Any disagreements between the reviewers were resolved based on pre-established criteria. Two independent reviewers then extracted data from the eligible articles using a standardized data extraction form created in Microsoft Excel. Any discrepancies during data extraction were resolved through discussion. The data that was extracted included the name of the first author, study area and region, study month and year, study design, year of publication, study population, sample size, response rate, and level of good knowledge, positive attitude, and poor practice.

### Quality assessment

To assess the quality of each study included in this systematic review, we used the modified Newcastle Ottawa Quality Assessment Scale (NOS) for cross-sectional studies [25]. Both authors (Chalachew Kassaw and Valeria Demareva) independently assessed the quality of each study, considering the following factors: methodological quality, sample selection, sample size, comparability of the study groups, outcome assessment, and statistical analysis. In the case of disagreement between authors, other reviewers were involved to resolve the issue. All studies included in this systematic review were cross-sectional, quantitative, or qualitative studies **(S3 File).**

## Results

### Study search and selection

**Fig 1:**
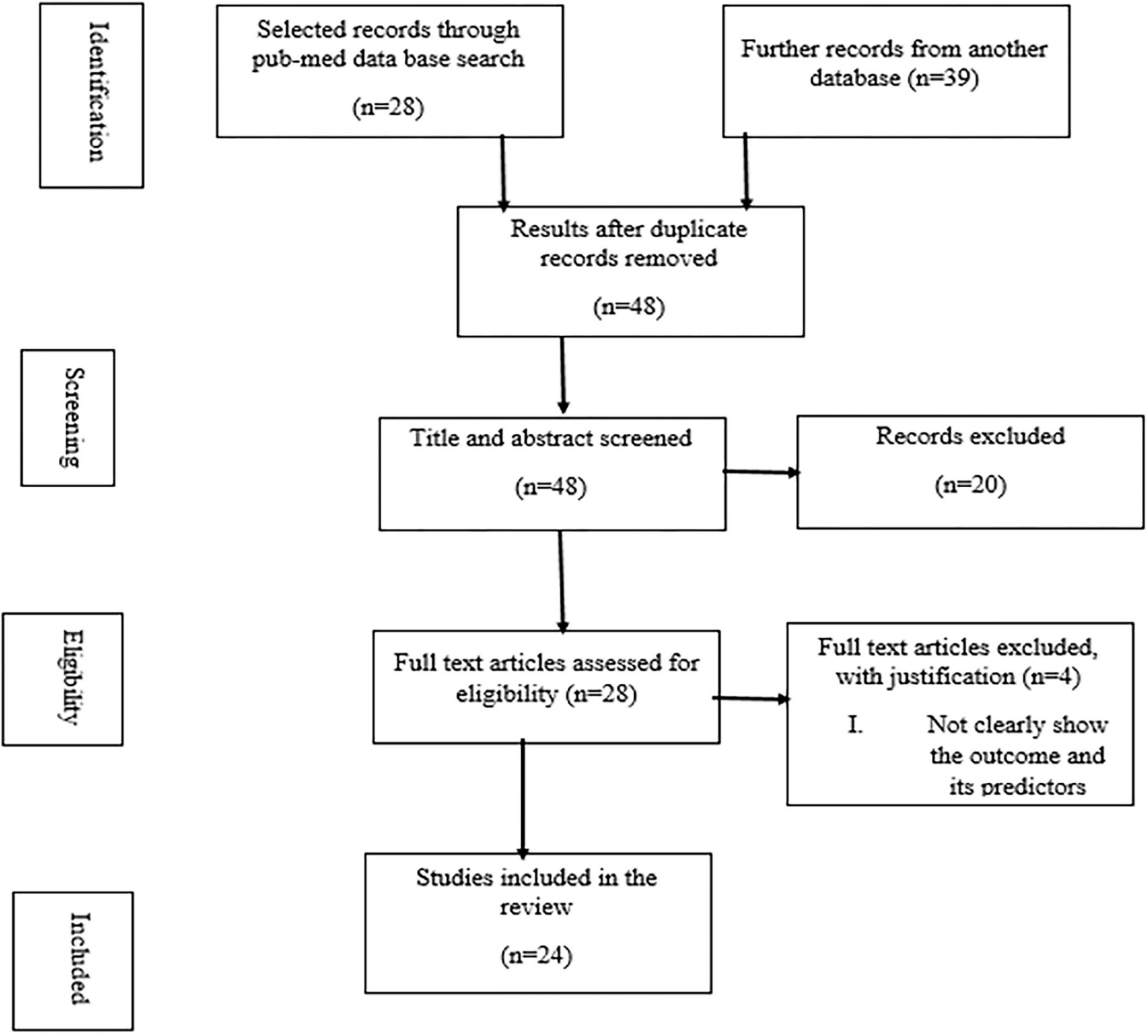
The flow chart that showed the selection process of papers for systematic review.

This study conducted a systematic review of academic achievement and its related factors by limiting the search to full-text articles in English published between 2013 and 2022 in the following databases: PubMed, Scopus, Google Scholar, African Journals Online, and Web of Science. A total of 67 primary papers were found, and 19 and 20 publications were discarded as duplicates and unrelated to the study, respectively, after title and abstract screening. Of the remaining 28 papers, four were excluded due to inadequate evidence of the relationship between academic achievement and its factors. Finally, 24 papers that met all inclusion requirements were selected for the systematic review. The rigorous methodology used in this study highlights the importance of selecting relevant papers to establish robust findings that can support subsequent research on academic achievement and its factors **(Fig 1).**

### Magnitude

Most systematic reviews of academic achievement and its factors among college students in Ethiopia focus on average grade point and performance evaluation indicators, adapting these metrics to their research goals and regional contexts [26–32] However, high-quality research on the topic in Ethiopia is scarce. This study analyzed eligible peer-reviewed papers from various Ethiopian colleges published between 2013 and 2022. Most of these studies were cross-sectional and included samples of both men and women from institutions. However, two studies used a cross-sectional, qualitative design [33,34]. Most of the studies we reviewed [26–32] examined sociodemographic factors such as age, gender differences, and monthly pocket money as potential contributors to academic achievement. However, a small proportion of studies, specifically those that examined the relationship between menstruation and academic success, included interviews with women [35,36]. This study review included participants from all part of the nation, aged 18–35 years with an average of 21.2 years. It found that factors such as biological, psychological, social, student-teacher interaction and lifestyle characteristics are predictive of academic achievement (Tables 1–4).

**Table 1:** Psychological related factors associated with academic achievement in low resource setting over the past 10 years (2013–2022), 2023.

Author	Study area	Objective	Population sampling	Quality assessment of the study (Newcastle Ottawa Scale)	Main finding
Lemma S et al [37]	Haramaya and Gondar University	Is there any association between sleep quality and academic performance	2173 (471 female and 1,672 male), Mean age = 21.6, Min = 20, Max = 23 years old	6	This study finding showed that good sleep quality, male, and advancement in year of study was associated with academic achievement. Sleep quality = β = −0.012, Std.Err = 0.004, t-test = −3.03, P-value < 0.002), Male = β = 0.2 74, Std.Err = 0.0 26, t-test = 10.63, P-value <0.001), 4^th^ year and above, β = 0.19, Std.Err = 0.040, t-test = 4.80, P-value <0.001)
Tessema T et al [32]	Hawassa health college	Mental distress and academic achievement	280(126 male, 138 female), Min age = 19, Min = 16, Max = 25	7	Students who had Grade Point Average of below 60% were 4.69 times more likely to be mentally distressed as compared to those students who scored Grade Point Average of greater or equal to 60% (AOR = 4.69; 95% CI, [2.46, 8.96).
Tesfahunegn T et al [31]	Aksum University	Mental distress and academic achievement	919(597 male, 322 female), Mean age = 21.5, Min = 20, Max = 27	6	Scoring lower grade (AOR = 1.51, 95% CI: 1.03, 1.61) were significantly associated with mental distress.
Dachew BA, et al [38]	Gondar university	Mental distress and Academic achievement	836(538 male, 298 female), Mean age = 20, Min = 18, Max = 23	7	Lower grade than anticipated (AOR = 2.07; 95% CI 1.51–2.83) were significantly associated with mental distress.
Asfaw H et al [39]	Haramaya university	Suicidal ideations and attempt and academic achievement	710(489 male, 221 female), Mean age = 22.7, Min = 18, Max = 32	8	Cumulative grade point average (AOR = 0.30, 95% CI: 0.18–0.49) was associated suicidal ideation.
Gidi N et al [40]	Jimma university	Academic achievement and low self-esteem (LSE)	422(279 male, 143 female), Mean age = 22, Min = 18, Max = 25	5	Those who reported to have poor academic performance were also more likely to have LSE AOR = 3.7 (95% CI, 1.3–10.0).
Amhare A et al [28]	Salale University	Perceived stress and academics achievement	402(228 male, 174 female), Mean age = 20.5, Min = 20, Max = 23	6	Perceived stress is significantly but negatively correlated with grade point average [r = -0.25 (-0.334, -0.153)]
Getahun A et al [27]	Wollo University	Pro-social behavior and academic achievement	111(50 male, 53 female), Mean age = not specified	6	There was statistically significantly association between students’ prosocial behavior and academic achievement (r -.219, p < 0.05).
Ahmed G et al [41]	Jimma university	Depression and academic achievement	556(360 male, 196 female), Mean age = 21.2, min =, 18 max = 35	5	Promoted academic performance (OR = 2.912, 95% CI; 1.063–7.975) were significantly associated with depression.
Tsegay L et al [42]	Addis Ababa university	Test anxiety and academic achievement	423(232 Male, 158 female), Mean age = 21.8, Min = 18, Max = 26	7	Test anxiety was associated with academic achievement Having low grade [AOR = 0.11, 95% CI: (0.044, 0.288)] independently predicts test anxiety.

**Table 2:** Biological related factors associated with academic achievement in low resource setting over the past 10 years (2013–2022), 2023.

Author	Study area	Objective	Population sampling	Quality assessment of the study (Newcastle Ottawa Scale)	Main finding
Tadese M et al [43]	Hawassa university	What are the determinants of academic performance	659(367 male, 248 female) Mean age = 21.6,Min = 18,Max = 29)	7	Students aged between 20 and 24 years (AOR = 0.43, 95% CI = 0.22–0.91), and medical/ health faculty (AOR = 2.46, 95% CI = 1.45–4.20) were significant associates of good academic performance. Students who didn’t smoke cigarettes were three times more likely to score good academic grades compared to those who smoke (AOR = 3.15, 95% CI = 1.21–7.30).
Gedefaw A et al [44]	Hawassa University	Psychoactive substance and self-reported academic performance	592(473 male,119 female), Mean age = 22.1, Min = 15, Max = 27	5	Students who had never used tobacco, alcohol, or khat after starting university were twice as likely to score a self-reported cumulative GPA above 3.0 (adjusted odds ratio 1.95, 95% confidence interval 1.25–3.02) and less likely to be delayed, have to re-sit an examination, or be warned (adjusted odds ratio 0.47, 95% confidence interval 0.29–0.77).
Mekonen T et al [30]	Wolaita Sodo University	Psychosocial predictors, Substance use (alcohol, tobacco, and khat) and academic performance	725(482 male, 243 female), Mean age = 21.1, Min = 20, Max = 23	7	The variables with significant association to academic performance were male gender (B = 0.35, 95% CI: 0.19, 0.52), urban origin of residence (B = −0.19, 95% CI: −0.32, −0.06),current smoking (B = −0.27, 95% CI: −0.46, −0.09), chewing khat at least weekly (B = −0.24, 95% CI: 0.44, −0.04), having intimate friend who uses substance (B = −0.17, 95% CI: −0.31, −0.03), and drinking alcohol on a daily basis (B = −0.51, 95% CI: −0.79, −0.23)
Tulu SK et al [34]	Mekelle university	Psychoactive substance use and Academic achievement	61(25 Male, 36 female), Mean age = 23, Min = 20, Max = 27)	7	Almost all participants 180(90%) are responded that they are experienced academic consequences that are resulted from alcohol and drug abuse.
Alemu S et al [35]	Debre Berhan University	Effect on Mental and reproductive health correlates of academic performance	529(529 female), Mean age = 20.5, Min = 19, Max = 23	8	There was a significant difference in academic performance between students with different length of menses (F-statistic = 5.15, p value = 0.006).
Kibwana S et al [45]	Six public universities	Factors affecting academic competencies	122(88 male,34 female),Mean age = 23, Min = 20, Max = 30	5	Male graduates significantly outscored female graduates overall (63.2% versus 56.9%, P = 0.014
Mesele T et al [36]	Haramaya university	The impact of dysmenorrhea on academic performance	356(356 female, Mean age = 22, Min = 18, Max = 28)	7	Premenstrual syndrome [AOR = 4.86:95%CI (2.13, 11.06)], early menarche [AOR = 4.89:95%CI (2.03, 11.77)], moderate/severe dysmenorrhea pain intensity [AOR = 8.53:95%CI (4.45, 16.39)], and students monthly pocket money <150ETB [AOR = 3.91:95%CI (1.48, 10.29)] were significantly associated with the occurrence of the impact of dysmenorrhea on academic performance.

**Table 3:** Facility and educational environment related factors associated with academic achievement in low resource setting over the past 10 years (2013–2022), 2023.

Author	Study area	Objective	Population sampling	Quality assessment of the study (Newcastle Ottawa Scale)	Main finding
Tiruneh S et al [46]	Bahir Dar University	What are facility-related factors affecting medical students’ academic performance	120(81 male, 39 female), Mean age = 20 min = 18, Max = 24)	5	Dormitory crowdedness (AOR 3.16 (95% CI: 0.83–2.01, p = 0.11), large class size (AOR = 2.36; 95% CI: 1.11–4.64 p = 0.005), inadequate classroom facilities (AOR = 1.56; 95% CI: 1.51–4.91, p = 0.001), low internet access (AOR = 1.99; 95% CI: 1.07–3.22, p = 0.015) and inadequate anatomy-teaching model (AOR = 2.63; 95% CI: 1.17–6.12, p = 0.003) were significantly associated with low performance of students in human anatomy course exam
Mulualem Y et al [26]	Debere Markos university	Previous English achievement and academic performances.	473 (340 male, 133 female), Mean age = Not clear	8	Past English achievement had significant direct and indirect effects on English test scores and semester grade point average (SGPA) at college (r = 0.61)
Negash T et al [47]	Gondar and Debre Tabor University	The relation between educational environment and academic achievement	123 (91 male, 32 female), Mean age = 22, Min = 20, Max =, 24	7	Entrance exam result, students’ perception of teachers, students’ academic self -perception and students’ social self-perception showed positive correlation with students’ academic achievement (ß = 0.003 & P = 0.04, ß = 0.009 & P = 0.9, ß = 0.06 & P = 0.42, ß = 0.06 & P = 0.39, ß = 0.14 & P = 0.015 and ß = 0.13 & P = 0.023)

**Table 4:** Life style related factors associated with academic achievement in low resource setting over the past 10 years (2013–2022), 2023.

Author	Study area	Objective	Population sampling	Quality assessment of the study (Newcastle Ottawa Scale)	Main finding
Akibu M et al [33]	Debre Berhan University	Effect of Premarital sexual practice on Academic performance	604 (316 male, 288 female), Mean age = 22, Min = 18, Max = 25)	6	Having higher academic performance was found to be a protective factor against pre-marital sexual practice (AOR = 0.43; 95% CI; 0.25–0.74, P< 0.01).
Lake Y et al [48]	Dire-Dawa Polytechnic college	Social media usage, psychosocial wellbeing, and academic performance.	204 (126 male, 78 female), un-specified age group	6	Academic performance of students negatively correlated and significantly with social media usage (telegram (r = -0.25), Facebook(r = —.29), and What’s-app (r = -.38)
Gelibo T et al [49]	Wolaita Sodo University	Abstain from sexual inter course and academic achievement	750 (583 male, 166 female) Mean age = 20, Min = 18, Max = 22	6	Academic achievement was associated abstain from sexual intercourse AOR = 2.9 (1.9,4.6), P<0/05).

### Psychological predictors of academic achievement

According this study review result, sleep quality, mental distress, suicidal ideation, perceived stress, low self-esteem, depression, pro-social behavior and test anxiety were identified factors associated with academic achievement (Table 1).

### Biological predictors of academic achievement

Gender difference (male), age difference (20–24 years old), psychoactive substance use and menstrual related factors (dysmenorrhea and long menses period) were associated with academic achievement (Table 2).

### Facility and educational environment predictors of academic achievement

This study review revealed that Dormitory crowdedness, inadequate anatomy-teaching model, low internet access, Past English achievement, Entrance exam result, students’ perception of teachers, students’ academic self -perception and students’ social self-perception were associated with academic achievement (Table 3).

### Life style predictors of academic achievement

Student’s life style factors such as pre-marital sex, sexual abstinence and excessive social media (Facebook, What up and telegram) use were associated with academic achievement (Table 4).

### Summary of predictors of academic achievement

**Fig 2:**
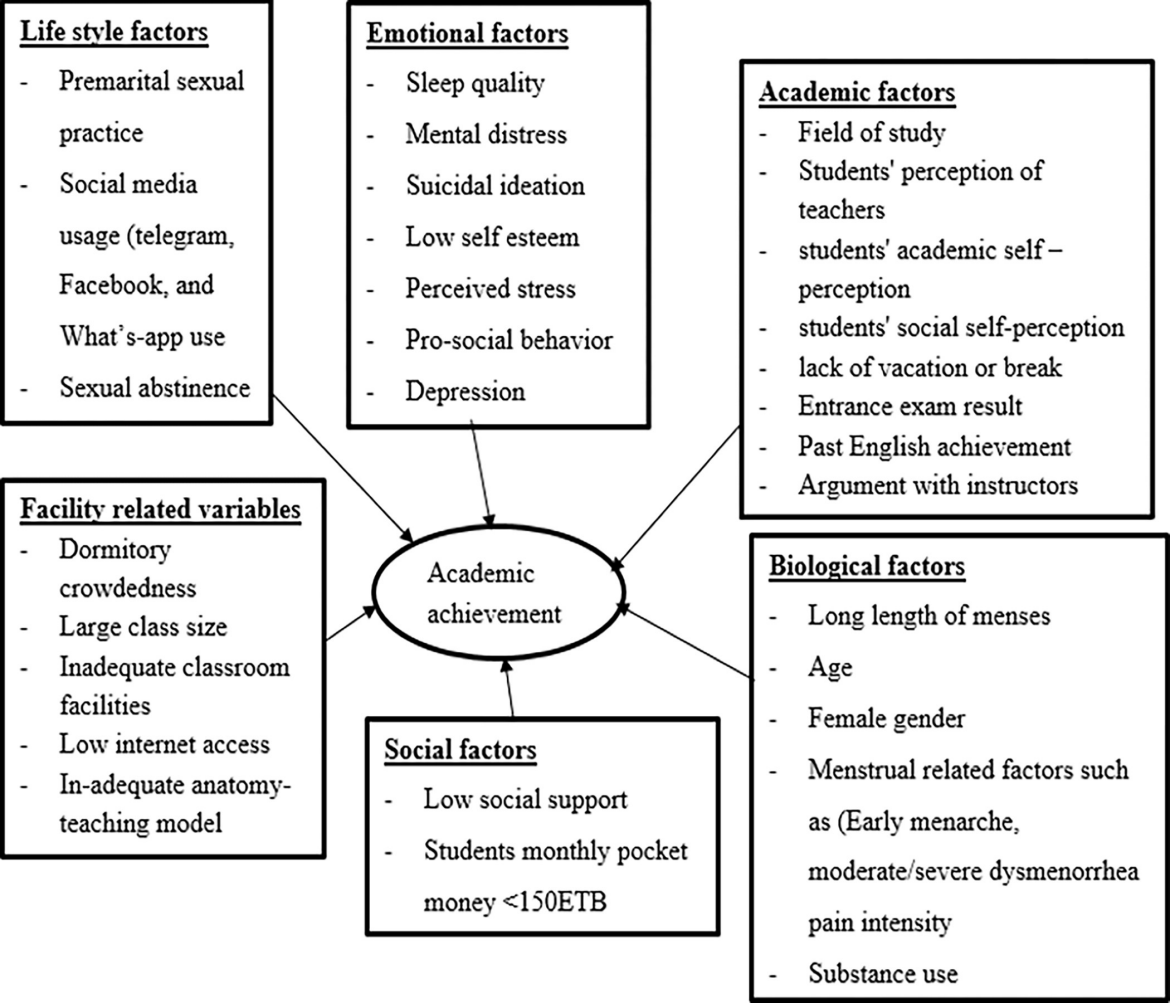
Summarized category of factors associated with academic achievement in higher education.

Several studies have found a significant negative correlation between academic achievement and factors. This include facility related factors such as large class sizes, insufficient internet access, poor classroom amenities, and teaching methods. Lifestyle style related factors such as excessive social media usage, premarital sex, and sexual abstinence have also been shown to have an impact on academic achievement. Psychlogical factors such as perceived stress, a lack of social support, and low self-esteem have also been found to influence academic success. Finally, Biological factors such as gender, age above 24, and hormonal issues, especially dysmenorrhea, are other factors that have been shown to have a significant impact on academic performance (Fig 2).

## Discussion

This systematic review explored the factors affecting academic success in Ethiopian higher education. Both modifiable and non-modifiable characteristics were identified as predictors of academic achievement.

### Socio-economic factors

This study review found that individuals with low socioeconomic status, specifically low monthly income and inadequate social support, were more likely to experience poor academic performance. This review result is similar with a systematic review done in Belgium [50], Pakistan [51], and Netherlands [52]. Financial constraints can prevent students from accessing basic educational tools, such as pencils, paper, laptops, and notebooks, which are necessary to attend classes efficiently and achieve good academic results. Students with strong social support are highly motivated to succeed in their studies, cope with challenges, manage stress and anxiety, and have opportunities to collaborate with others, discuss ideas, and get feedback on their work. Providing daily necessities such as financial and emotional support is essential for students’ physical and mental well-being.

### Biological factors

The study also found that biological factors, such as gender, age, and menstrual cycle-related hormone changes, are associated with academic success. These findings are consistent with research conducted in low- and middle-income countries [53] and Saudi Arabia [54] and China [55]. Advanced age may cause changes in all parts of the body, including the brain. Certain parts of the brain shrink, especially those that are important for learning and other complex mental processes [56]. Neuronal transmission may become less efficient in some areas of the brain as people age, which can lead to a decline in working memory and make tasks like making decisions and resolving problems more challenging [57]. Students with dysmenorrhea may miss classes, assignments, and tests, be reluctant to participate in class discussions or activities, experience pain and discomfort during class and study, and find it difficult to focus and concentrate, all of which can lead to lower academic achievement.

Estrogen increases the production of acetylcholine, a brain enzyme essential for memory, and strengthens neuronal connections in the hippocampus, a region of the brain critical for language recall. Estradiol, a hormone prevalent in women, is particularly important for word memory, focus, and rapid information processing [58,59].

### Psychological factors

This study review found that mental and psychological conditions such as melancholy, anxiety, suicidal thoughts, low self-esteem, perceived stress, prosocial behavior, and sleep disorders are important indicators of academic achievement in higher education. This finding was consistent with a research conducted in Italy [60], Australia [61], Nepal [62] and China [63]. Mental health issues can negatively influence students’ academic success by reducing their energy, focus, motivation, cognitive abilities, and optimism. Depressed or anxious students may find it difficult to socialize and participate in class, which can lead to a decline in their academic performance. Students struggling with mental health concerns may also become less engaged and proactive in their studies [64,65].

### Life style factors

The systematic review found that premarital sex, social media use, and sexual abstinence could affect a person’s way of life. The same conclusion was reached in a Latin American study [66] and China [67]. Students who engage in premarital sex may be more likely to experience academic failure for a number of reasons. They may spend more time with their partners, miss more classes, and get distracted. They may also feel guilty, low self-esteem, and susceptible to physical illnesses. All of these factors can contribute to academic problems [68]. A major drawback of technology is that social media use can distract students from their academic work. When students are bombarded with both educational and entertainment messages, it can be difficult for them to concentrate on their lectures. Additionally, students may prioritize online chatting and building relationships on social media over reading books in their free time, which can further harm their academic performance [69,70].

This study also found that certain facility-related factors, such as crowded dorm quarters, large class sizes, inadequate classroom amenities, and restricted internet access, are associated with academic achievement. This result was supported with a study done United Kingdom [71], Malaysia [72] and Korea [73]. This may be explained by inadequate school infrastructure, which can distract, tire, and disengage students, making it difficult for them to learn effectively. Examples of poor school facilities include loud noises, crowding, poor lighting, and difficulty accessing instructional materials [74].

This review found that the field of study, a good student-teacher relationship, the absence of breaks, and the performance of previous students are all academically linked criteria, consistent with reviews and studies conducted in the United Kingdom [75], Unites States of America [76], Nepal [77] and China [78]. Students are more likely to study when they feel positive about their learning environment. This is because they are more motivated to learn when they feel a sense of belonging, competence, and autonomy in their academic setting [79]. Factors in your classroom environment can influence student motivation. Motivated students put more effort into learning activities, such as paying attention, overcoming challenges, interacting with others, forming friendships, and managing their emotions (e.g., sadness and anxiety) [80].

### Strengths of the study review

The review searched five databases to retrieve relevant articles.

The review strictly followed PRISMA flow charts.

More than one assessor evaluated the quality of the studies.

The review used the appraisal process developed by the Joanna Briggs Institute (JBI).

The review included studies from all parts of the country, ensuring good representativeness.

### Limitations of the study review

The measurements for academic achievement and operational definitions may have differed between the primary studies.

### Conclusion

This systematic review analyzed the predictors of academic achievement in Ethiopian higher education students, as identified in primary studies conducted over the past 10 years. The review identified many factors that affect academic achievement, including controllable factors such as facility-related variables, emotional factors, and lifestyle factors. These include large class sizes, poor internet connections, inadequate classroom facilities, poor teaching strategies, perceived stress, lack of social support, and substance use.

### Recommendation

Based on the findings of this systematic review, it is recommended that universities and colleges in Ethiopia take steps to improve facility-related resources, provide support for student emotional and mental well-being, educate students about healthy lifestyle choices, and develop and implement interventions to address specific predictors of academic achievement.

Improving facility-related resources includes reducing class sizes, improving internet access, and providing adequate classroom facilities and teaching materials. This can help to create a more conducive learning environment for students and support their academic success.

Providing support for student emotional and mental well-being can be done by offering counseling services, creating a supportive campus environment, and raising awareness of the importance of mental health. This can help to reduce stress and anxiety among students, which can improve their academic performance. Educating students about healthy lifestyle choices can be done through workshops, seminars, and other educational programs. This can help students to make informed decisions about their health and well-being, which can indirectly lead to improved academic achievement. Developing and implementing interventions to address specific predictors of academic achievement can involve a variety of strategies. For example, interventions could be designed to reduce stress, improve social support, and prevent substance use. These interventions can be tailored to the specific needs of the student population and can be delivered in a variety of settings, such as classrooms, residence halls, and student health centers. By taking these steps, universities and colleges in Ethiopia can help to improve the academic achievement of their students and create a supportive and inclusive learning environment.

## Supporting information

S1 File Preferred Reporting Items for Systematic Reviews and Meta-Analyses (PRISMA) guideline. (DOCX)

S2 File Search strategy. (DOCX)

S3 File Newcastle-Ottawa Quality assessment scale. (DOCX)

S4 File Microsoft excel document. (ODS)
