# Supplementary material for: Determinants of academic achievement among higher education student found in low resource setting, A systematic review
Source: PLoS One. 2023 Nov 20;18(11):e0294585. doi: 10.1371/journal.pone.0294585 (PMC10659171; doi:10.1371/journal.pone.0294585)
Supplement: S1 File — (DOCX) [file pone.0294585.s001.docx]

**S1 Table. Prisma checklist**

| **Item** | | **Criteria to score as adequately reported** |
| --- | --- | --- |
| *Title* | | |
| 1 | Title | The title contained *systematic review* and/or *meta-analysis*.^$^ |
| *Background* | | |
| 2 | Objectives | The objective of the study with reference to Patients, Interventions, Comparisons, Outcomes and Study design (PICOS) was reported. |
| *Methods* | | |
| 3 | Eligibility criteria | Study characteristics (PICOS) and report characteristics used as criteria for inclusion were specified. |
| 4 | Information sources | Information sources and date of last search were described. |
| 5 | Risk of bias | Methods to assess risk of bias were reported. |
| *Results* | | |
| 6 | Included studies | Number and type of included studies and participants were reported. |
| 7 | Synthesis of results | Results for main outcomes were presented. |
| 8 | Description of the effect | Direction and size of the effect was reported. |
| *Discussion* | | |
| 9 | Strengths and Limitations of the evidence | Strengths and limitations of the evidence were discussed. |
| 10 | Interpretation | Results and implications were interpreted. |
| *Other* | | |
| 11 | Funding | Source of funding for the review was stated. |
| 12 | Registration | Registry name and registration number was provided. |

From: Moher D, Liberati A, Tetzlaff J, Altman DG, The PRISMA Group (2009). Preferred Reporting Items for Systematic Reviews and Meta-Analyses: The PRISMA Statement. PLoS Med 6(7): e1000097. doi:10.1371/journal.pmed1000097 For more information, visit: [www.prisma-statement.org](http://www.prisma-statement.org).
