# Supplementary material for: Determinants of academic achievement among higher education student found in low resource setting, A systematic review
Source: PLoS One. 2023 Nov 20;18(11):e0294585. doi: 10.1371/journal.pone.0294585 (PMC10659171; doi:10.1371/journal.pone.0294585)
Supplement: S2 File — (DOCX) [file pone.0294585.s002.docx]

**S2 Table. Searching strategy**

| Search engine | “Academic Achievement”, OR “Academic Performance” OR “Average Cumulative Grade Point”, OR “Performance Indicators” AND Psychological Determinants”, “Biological Determinants”, “Social Determinants”, “Higher Education”, “Competency Measures”, AND “Teaching-learning styles Predictors” AND “Ethiopia” | | Number of articles |
| --- | --- | --- | --- |
| Pub-med |  | | 15 |
| Scopus |  | | 6 |
| African Journals Online |  | | 7 |
| Web of Sciences |  | | 5 |
| Google scholar |  | | 31 |
| Public library science |  | | 3 |
| Total |  | | 67 |
| Excluded due different reason | Reason one: - Screened by title/abstract/ Not English and Not clearly state psychosocial determinants academic performance (n=10).  Reason Two:- Not explicitly state a facility , social, psychological, biological and teaching-learning methods related predictors of academic performance/Not primary studies/Updated studies available ( n=33). | | 43 |
| Included studies for this review | |  | 24 |
