# Supplementary material for: Determinants of academic achievement among higher education student found in low resource setting, A systematic review
Source: PLoS One. 2023 Nov 20;18(11):e0294585. doi: 10.1371/journal.pone.0294585 (PMC10659171; doi:10.1371/journal.pone.0294585)
Supplement: S3 File — (DOCX) [file pone.0294585.s003.docx]

**S3 Table. Quality assessment of the study**

| Assessment Items | Scoring |
| --- | --- |
| Representativeness of the sample | a) Truly representative of the average in the target population. * (all subjects or random sampling)  b) Somewhat representative of the average in the target population. * (non-random sampling)  c) Selected group of users. (0)  d) No description of the sampling strategy.(0) |
| Sample size | a) Justified and satisfactory. *  b) Not justified. (0) |
| Non-respondents | a) Comparability between respondents and non-respondents’ characteristics is established, and the response rate is satisfactory. (*)  b) The response rate is unsatisfactory, or the comparability between respondents and non-respondents is unsatisfactory. (0)  c) No description of the response rate or the characteristics of the responders and the non-responders.(0) |
| Ascertainment of the exposure (risk factor) | a) Validated measurement tool. **  b) Non-validated measurement tool, but the tool is available or described.*  c) No description of the measurement tool. |
| Comparability:  (Maximum 2 stars) | 1) The subjects in different outcome groups are comparable, based on the study design or analysis. Confounding factors are controlled.  a) The study controls for the most important factor (select one). *  b) The study control for any additional factor. * |
| Outcome: (Maximum 3 stars) | 1) Assessment of the outcome:  a) 6) Independent blind assessment. **  b) Record linkage. **  c) Self report. *  d) No description.  2) Statistical test:  a) The statistical test used to analyse the data is clearly described and appropriate, and the measurement of the association is presented, including confidence intervals and the probability level (p value). *  b) The statistical test is not appropriate, not described or incomplete |

*; 1 point, **; 2 point***; 3 point
